# Application of Beneficial Bacteria to Enhance Plant Drought Resilience

**DOI:** 10.3390/plants15050753

**Published:** 2026-02-28

**Authors:** Yryszhan Zhakypbek, Bekzhan D. Kossalbayev, Serik Tursbekov, Galiya Tursbekova, Zhansulu Berdaliyeva, Ayaz M. Belkozhayev

**Affiliations:** 1 Department of Mine Surveying and Geodesy, Mining and Metallurgical Institute Named After O.A. Baikonurov, Satbayev University, Almaty 050043, Kazakhstan; y.zhakypbek@satbayev.university; 2 Department of Chemical and Biochemical Engineering, Geology and Oil-Gas Business Institute Named After K. Turyssov, Satbayev University, Almaty 050043, Kazakhstan; 3 Ecology Research Institute, Khoja Akhmet Yassawi International Kazakh Turkish University, Turkistan 161200, Kazakhstan; 4 Faculty of Biology and Biotechnology, Al-Farabi Kazakh National University, Al-Farabi Ave. 71, Almaty 050038, Kazakhstan; 5 Department of Engineering Biochemistry, International Engineering-Technological University Kazakhstan, Almaty 050060, Kazakhstan; tursbekova1956@mail.ru; 6 Department of Geodesy, Cartography and Cadastre, Kazakh Head Architecture and Construction Academy, Almaty 050043, Kazakhstan; zhberdaliyevazh@gmail.com; 7 M.A. Aitkhozhin Institute of Molecular Biology and Biochemistry, Almaty 050012, Kazakhstan

**Keywords:** plant drought stress, plant growth-promoting bacteria (PGPB), rhizosphere microbiome, phytohormone regulation, ABA signaling, osmotic adjustment, antioxidant defense, root architecture

## Abstract

Drought stress is one of the most severe abiotic constraints limiting crop productivity worldwide, a challenge that is intensifying under ongoing climate change. In recent years, beneficial microorganisms have emerged as sustainable, nature-based tools to enhance plant drought tolerance and stabilize agricultural production under water-limited conditions. This review synthesizes current knowledge on the major groups of beneficial bacteria involved in drought stress mitigation, including plant growth-promoting rhizobacteria (PGPR), a functional subgroup of rhizosphere-associated microbes, endophytic bacteria, rhizosphere-associated microbes, and cyanobacteria, highlighting their primary physiological, biochemical, and soil-mediated mechanisms. These microorganisms enhance drought resilience through multiple complementary pathways, such as modulation of abscisic acid (ABA) and auxin (IAA) signaling, ACC deaminase activity, osmotic adjustment, antioxidant defense, improved nutrient acquisition, and enhancement of soil structure and water retention. The review further discusses practical application strategies, including seed inoculation, soil and root application, foliar spraying, the use of single strains versus microbial consortia, and advances in bioformulations and carrier materials that improve microbial survival and field efficacy. Emphasis is placed on recent experimental and field studies demonstrating the effectiveness of microbial inoculants under drought conditions. Collectively, the evidence highlights the potential of beneficial bacteria as key components of climate-resilient agriculture and underscores the need for integrated, formulation-driven approaches to translate laboratory success into consistent field performance.

## 1. Introduction

Drought stress is one of the most serious abiotic factors threatening agricultural production worldwide [1,2]. Ongoing climate change, accompanied by reduced precipitation and rising global temperatures, has a negative impact on plant growth and development, ultimately leading to significant yield losses [3]. At the global scale, a substantial proportion of crop yield losses is directly or indirectly linked to drought conditions [4,5]. In many arid and semi-arid regions, the frequency and intensity of drought events are projected to increase further, posing an even greater challenge to global food security [6]. Water deficiency suppresses photosynthetic activity, disrupts cellular osmotic balance, limits the uptake of essential nutrients, and causes damage to cellular structures [7]. At the cellular level, drought stress alters membrane stability, enzyme activity, and metabolic fluxes, leading to impaired growth and reduced biomass accumulation [1,7]. As a result, drought stress weakens plant tolerance mechanisms at both physiological and molecular levels. To mitigate the negative effects of drought stress, a range of conventional approaches has been widely applied, including improvements in irrigation practices, the use of chemical fertilizers and plant growth regulators, as well as the development and breeding of drought-tolerant crop varieties [8,9]. These strategies have contributed to yield stabilization in certain environments; however, their effectiveness strongly depends on local climatic, soil, and management conditions. These approaches have several limitations, including limited water availability, high economic costs, increased environmental burden, and, in some cases, reduced long-term effectiveness [10]. Excessive reliance on chemical inputs may further exacerbate soil degradation and negatively affect agroecosystem sustainability [11]. In addition, genetic breeding and biotechnological strategies require substantial time and financial investment, and their outcomes are not always consistent across different environmental conditions [12]. Similarly, although microbial-based approaches are increasingly regarded as sustainable alternatives, their field performance can also be influenced by environmental variability, soil properties, host specificity, and formulation stability, which may limit consistent outcomes across agroecosystems [13]. In recent years, increasing attention has been given to the use of beneficial microorganisms, particularly plant growth-promoting bacteria (PGPB), to enhance plant tolerance to drought stress [14]. These bacteria enhance plant adaptation by modulating phytohormone balance, reducing ethylene levels, activating stress-protective systems, and improving nutrient availability [15,16]. Importantly, PGPB-mediated responses often involve the coordinated regulation of antioxidant defense, osmotic adjustment, and stress-responsive gene expression [15]. The application of beneficial bacteria is therefore considered an environmentally friendly, cost-effective, and sustainable alternative for improving agricultural resilience [14,15]. Such microbial-based strategies are increasingly recognized as key components of climate-smart and sustainable agriculture [17]. This review provides a comprehensive and critical synthesis of plant physiological, biochemical, and molecular responses to drought stress, as well as the role of beneficial bacteria in enhancing plant drought tolerance. It covers key processes including plant water relations, photosynthesis, growth, reactive oxygen species (ROS) production, osmolyte accumulation, antioxidant defense systems, and drought-responsive hormonal and genetic regulation.

## 2. Plant Responses to Drought Stress

### 2.1. Physiological Responses

Drought stress imposes immediate physiological challenges on plants by disrupting water relations and reducing carbon assimilation. Limited soil moisture leads to lowered plant water potential and stomatal closure, which conserves water but also restricts CO_2_ uptake and photosynthesis [18,19]. As drought progresses, plants exhibit reduced growth rates, biomass accumulation, and leaf expansion [1,10]. Many drought-sensitive species show marked declines in relative water content (RWC) of tissues, wilting, and impaired photosynthetic efficiency, ultimately translating into diminished yields [20]. Some plants can partially maintain physiological function via adaptive traits, but severe or prolonged water deficits invariably strain fundamental processes like photosynthesis and growth across diverse species [21]. Khamis et al. (2025) studied wheat and amaranth under drought. Drought markedly decreased leaf water status, net photosynthetic rate, and dry mass in both, with wheat showing greater growth inhibition than amaranth [22]. Photosynthetic decline was linked to stomatal closure and impaired PSII function (lower Fv/Fm), while amaranth’s physiology conferred a bit more resilience. This paper illustrates how drought disrupts water balance and photosynthesis, with severity depending on plant type. Consistent with these findings, Dong et al. (2025) examined six plant species from arid northwestern China under controlled drought gradients and observed significant reductions in height growth and leaf mass per area across all species [23]. Leaf water content declined progressively with increasing drought severity; however, species such as *Nitraria* and *Haloxylon* retained higher leaf water status compared with *Tamarix* and *Potentilla* [23]. These results highlight both common drought-induced growth suppression and species-specific differences in water retention capacity. These physiological responses to drought are further reflected in agronomically important crop species under controlled water deficit conditions. A pot experiment on broccoli cultivars under four irrigation levels. Well-watered plants (100% field capacity) had highest leaf number, plant height, and yield, while severe drought (45% FC) cut yield by ~40% [24]. Drought-stressed broccoli showed fewer leaves, shorter stature, and lower chlorophyll content, indicating reduced growth and photosynthetic activity. Interestingly, water deficit elevated certain quality traits as stress responses, but overall growth and yield were negatively affected. It has been widely reported that drought-induced reductions in photosynthesis result from limitations affecting both the light-dependent and carbon fixation reactions. Water deficit impairs chloroplast electron transport and ATP production, while stomatal closure restricts CO_2_ availability and inhibits key enzymes of the Calvin cycle, ultimately leading to reduced growth and yield in major crops such as wheat, rice, and maize [25]. In addition to these photosynthetic constraints, drought stress also triggers protective physiological adjustments. Experimental evidence from woody species indicates that drought suppresses growth while inducing the accumulation of osmolytes and enhancing antioxidant enzyme activity as adaptive responses [1,23]. Moderate drought effects can be partially reversible upon rewatering; however, severe or prolonged stress often causes persistent physiological damage and incomplete recovery of growth, highlighting the importance of stress intensity and duration in determining plant resilience [26]. Drought-induced disruption of plant water status leads to stomatal closure, reduced photosynthesis, and growth inhibition, with response severity depending on species traits and stress intensity [18,25]. These physiological changes provide the foundation for subsequent biochemical and molecular stress responses.

### 2.2. Biochemical Responses

Drought triggers a cascade of biochemical changes in plant cells. A prominent effect is the overproduction of ROS such as superoxide and hydrogen peroxide due to metabolic imbalances under water deficit [27]. To counteract drought-induced ROS damage to membranes, proteins, and DNA, plants upregulate enzymatic and non-enzymatic antioxidant systems to scavenge ROS and protect cellular integrity [28]. Concurrently, plants accumulate compatible osmolytes low-molecular-weight solutes like proline, glycine betaine, soluble sugars which help maintain cell turgor and protect proteins during dehydration. These osmolytes contribute to osmotic adjustment, stabilizing membranes and enzymes [29,30]. Drought-induced biochemical responses thus center on mitigating oxidative stress and osmotic stress: resilient plants balance ROS production with antioxidant defense and build up osmoprotectants to endure water loss. Hou et al. (2021) investigated drought tolerance mechanisms in the xerophytic sedge *Carex duriuscula*. Drought stress induced a marked increase in H_2_O_2_ and O_2_^−^ levels in both leaves and roots, indicating enhanced oxidative stress. Concurrently, antioxidant enzyme activities, including superoxide dismutase (SOD), peroxidase (POD), and ascorbate peroxidase (APX), were strongly upregulated, particularly under severe drought conditions, contributing to ROS detoxification. In addition, plants accumulated organic osmolytes such as soluble sugars and proline, as well as inorganic ions (K^+^, Na^+^, and Cl^−^), supporting osmotic adjustment [31]. Field evidence shows that drought enhances oxidative damage markers (MDA, H_2_O_2_) while inducing phenolics, flavonoids, peroxidase activity, and proline accumulation, with tolerant genotypes exhibiting stronger antioxidant responses linked to improved drought performance [32]. Beyond genotypic variation, experimental manipulation studies further clarify the protective role of osmolytes. In maize, exogenous proline application significantly reduced drought-induced oxidative damage, lowered ROS and MDA accumulation, enhanced antioxidant enzyme activities, and improved osmotic balance, membrane stability, and growth under water deficit [33]. At the conceptual level, drought-induced oxidative stress is now recognized as a dual process involving both cellular damage and stress signaling. As emphasized in a comprehensive review by Nahar et al. (2020), drought tolerance depends on maintaining ROS at manageable levels through a tightly coordinated antioxidant system that balances detoxification with redox signaling, thereby preserving photosynthetic machinery and membrane integrity [34]. Extending this perspective across species, meta-analytical evidence indicates that proline accumulation represents a widespread biochemical response to drought, although its magnitude is shaped by species traits, seed characteristics, and stress duration. Kijowska-Oberc et al. (2023) showed that woody plants, particularly deciduous species and those derived from desiccation-tolerant seeds, exhibit stronger proline accumulation, supporting its role as a general biochemical marker of drought resilience while highlighting important ecological and evolutionary modulation [35]. Together, these findings indicate that effective drought tolerance relies on tight control of ROS homeostasis and coordinated osmolyte accumulation, which collectively stabilize cellular structures and metabolic functions under water deficit.

### 2.3. Molecular and Hormonal Regulation

At the molecular level, drought stress orchestrates complex changes in gene expression and hormone signaling pathways. A central player is the hormone abscisic acid (ABA), which accumulates rapidly during drought and acts as a global stress signal [36,37]. ABA triggers downstream responses such as stomatal closure and activates numerous ABA-responsive genes that enhance drought tolerance [38]. Key enzymes in ABA biosynthesis are transcriptionally induced under dehydration, raising ABA levels that modulate guard cell behavior and stress gene activation [39]. Drought also induces a suite of transcription factors that regulate stress-responsive genes. Many drought-inducible genes code for protective proteins: LEA/dehydrins, osmolyte synthesis enzymes, antioxidant proteins, aquaporins, and other functional proteins that mitigate stress damage (Table 1) [40].

**Figure 1:**
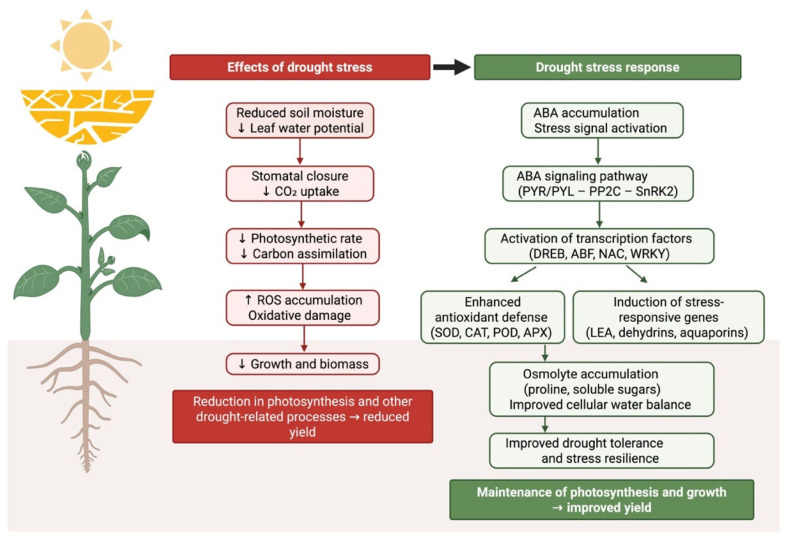
ABA-centered physiological, biochemical, and molecular responses of plants to drought stress. Created with BioRender.com (2026) by A.M. Belkozhayev (License No. BW29AAMTTV).

Concurrently, drought-repressed genes are often involved in growth and photosynthesis, reflecting a strategic reallocation of resources [40]. Hormonal crosstalk is integral to this process; in addition to ABA, signaling pathways involving ethylene, jasmonates, cytokinins, and other phytohormones interact to fine-tune gene regulatory networks and adaptive responses [50]. Drought ultimately triggers an ABA-centric regulatory network that coordinates hormone signaling and stress-responsive gene expression to adjust plant physiology. Together with the associated physiological and biochemical adjustments described above, these responses form an integrated drought-adaptation framework (Figure 1). At the molecular level, drought tolerance is largely governed by ABA-centered gene regulatory networks that integrate stress perception with transcriptional reprogramming. Comparative transcriptomic analyses in woody species have demonstrated that enhanced ABA biosynthesis is a key determinant of drought tolerance. In drought-tolerant poplar cultivars, genes encoding 9-cis-epoxycarotenoid dioxygenase (*NCED*), the rate-limiting enzyme in ABA biosynthesis, are strongly upregulated, leading to higher ABA accumulation during dehydration, whereas sensitive cultivars show suppressed *NCED* expression. Functional validation further confirms this mechanism, as *NCED*-deficient mutants exhibit extreme drought sensitivity, while *NCED* overexpression enhances stress tolerance through ABA-mediated gene regulation and stomatal control [51]. Beyond ABA biosynthesis, ABA-responsive transcription factors play critical roles in shaping stress outcomes at specific developmental stages. During seed germination, the bZIP transcription factor *ABI5* functions as a negative regulator of drought tolerance by enforcing ABA-dependent growth restraint. Genetic disruption of *ABI5* derepresses stress-responsive gene networks, alters ABA-related metabolic pathways, and promotes faster germination and root growth under water deficit, highlighting the stage-specific regulatory complexity of ABA signaling [52]. At the signaling-network level, drought perception triggers interconnected cascades involving calcium influx, ROS, and ABA accumulation, which collectively activate stress-responsive transcription factors and kinases [53]. Recent integrative reviews further emphasize that ABA-driven gene regulation is embedded within broader signaling frameworks that include ROS signaling, stress memory, and interactions with the root-associated microbiome. According to Ali et al. (2025), convergence of ABA and ROS signaling is central to guard cell regulation and transcriptional control, while beneficial microorganisms can modulate host ABA biosynthesis and antioxidant gene expression, thereby enhancing drought resilience [54]. At the genome-wide scale, drought-responsive gene networks encompass thousands of genes that can be broadly classified into functional protectors and regulatory orchestrators [55]. As reviewed by Nakashima et al. (2025) [45], ABA-dependent regulons dominate drought-induced transcriptional responses, including genes involved in ABA biosynthesis, ABA-responsive transcription factors (DREB, AREB/ABF), stress-activated kinases, and downstream protective proteins such as LEA proteins, osmolyte biosynthetic enzymes, detoxification enzymes, and aquaporins. Conversely, drought represses genes associated with growth and photosynthesis, reflecting an energy-saving strategy that prioritizes survival over biomass accumulation. Together, these findings underscore that drought tolerance emerges from tightly coordinated ABA-centered gene networks that dynamically balance growth restraint and stress protection.

## 3. Mechanisms of Bacteria-Induced Drought Tolerance

### 3.1. Phytohormonal Regulation and Signaling

Drought stress disrupts plant hormone balance and restricts growth [56]. Beneficial bacteria improve drought tolerance by regulating phytohormone signaling, including auxins, cytokinins, and ethylene, thereby promoting root development, stomatal control, and stress-responsive gene activation under water deficit [57,58,59]. This hormone-mediated mechanism has been supported by experimental studies demonstrating the role of phytohormone-producing bacteria in improving plant performance under drought. Uzma et al. (2022) demonstrated that indole-3-acetic acid (IAA)-producing *Pseudomonas* strains conferred drought tolerance in mung bean. Inoculated plants showed significantly improved growth and yield under water deficit, with up to ~293% increase in yield over drought controls. The IAA-producing bacteria enhanced root development and stress metabolite accumulation, highlighting hormonal modulation as a key mechanism [59].

Further evidence indicates that bacterial regulation of plant hormone signaling plays a central role in drought adaptation. Cytokinin-producing *Pseudomonas fluorescens* strains have been shown to prime tomato plants for drought by enhancing ABA accumulation, promoting stomatal closure, maintaining chlorophyll content, and inducing drought-responsive gene expression; loss of bacterial cytokinin production markedly reduced these protective effects, confirming the importance of microbial cytokinins in stress signaling [60].

In addition to auxin- and cytokinin-mediated effects, modulation of ethylene signaling represents another major phytohormonal pathway through which beneficial bacteria enhance drought tolerance. Zarei et al. (2020) reported that inoculation with ACC deaminase producing *Pseudomonas fluorescens* strains significantly improved sweet corn performance under drought conditions. By lowering stress-induced ethylene levels, bacterial treatment enhanced chlorophyll content, osmotic adjustment, and grain yield compared with uninoculated controls. Combined bacterial inocula resulted in substantial yield improvements under limited irrigation, highlighting the importance of ethylene regulation by beneficial bacteria in mitigating drought stress [61].

**Figure 2:**
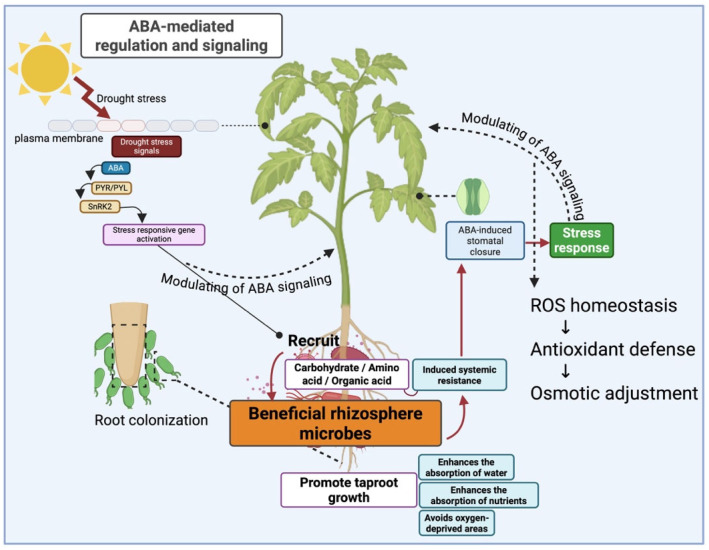
Bacterial regulation of plant ABA biosynthesis and signaling during drought. Created with BioRender.com (2026) by A.M. Belkozhayevwith BioRender.com (2026) by A.M. Belkozhayev (License No. MK29AF0G5L).

Beyond classical phytohormones, bacterial volatile organic compounds have also been implicated in drought tolerance, as *Bacillus pseudomycoides* VOCs were shown to modulate plant transcription factors, inducing DREB-mediated stress responses and enhancing growth, water status, and antioxidant activity in wheat [62]. These studies demonstrate that phytohormonal regulation represents a central and integrative mechanism by which beneficial bacteria enhance plant drought tolerance. By fine-tuning auxin, cytokinin, ethylene, and ABA signaling, as well as hormone-linked transcriptional pathways, plant growth-promoting bacteria coordinate root development, stomatal behavior, and stress-responsive gene expression, thereby improving plant performance under water-limited conditions across diverse crop species [63]. These integrated bacteria–phytohormone interactions, including bacterial stimulation of ABA biosynthesis and activation of ABA-responsive genes, are summarized in Figure 2.

### 3.2. Root Architecture Remodeling and Nutrient Acquisition

Drought resilience is strongly tied to root system architecture and nutrient status [64]. Beneficial bacteria stimulate root growth and branching, leading to deeper or more extensive roots that explore soil moisture better (Table 2) [64,65]. Many PGPB also enhance nutrient acquisition under drought through nitrogen fixation, phosphate solubilization, or siderophore production ensuring plants remain nourished when water stress limits nutrient mobility. By reshaping root morphology and improving nutrient uptake, bacterial inoculants help plants maintain water uptake and metabolic function during drought [66,67,68].

Fonseca et al. (2022) demonstrated that inoculation of sugarcane with *Bacillus subtilis* significantly enhanced root architecture under drought stress. Treated plants developed longer roots, increased tiller number, and higher root biomass, which was accompanied by improved nitrogen and phosphorus uptake [69]. Enhanced nutrient acquisition supported higher photosynthetic performance, water-use efficiency, and ultimately greater stalk weight and sucrose yield despite water limitation, underscoring the functional link between root remodeling and drought resilience.

Similarly, Almeida et al. (2024) reported that co-inoculation with native Bacillus strains substantially improved root surface area and root volume in chili pepper seedlings exposed to drought. Bacteria-treated plants exhibited markedly higher uptake of nitrogen, phosphorus, and micronutrients (Zn, Mn, Cu), which translated into increased shoot biomass, improved survival, and enhanced reproductive development under water deficit [70]. These findings highlight how bacterial enhancement of root absorptive capacity supports both vegetative growth and yield-related traits during drought.

In cereal crops, root system modification plays an especially critical role in drought avoidance. Noureen et al. (2024) found that drought-tolerant *Bacillus* strains, particularly when combined with biochar, markedly increased root length and depth in wheat under drought. Enhanced root penetration into deeper soil layers improved water uptake, resulting in higher relative water content, shoot growth, and grain yield compared with uninoculated plants [71]. This study illustrates how bacterial-driven root elongation directly enhances access to deep soil moisture under drought conditions.

At a broader conceptual level, Ahmad et al. (2022) emphasized that bacterial-induced root remodeling represents a conserved and central mechanism of drought tolerance across plant species. Through auxin production and ACC deaminase activity, beneficial bacteria stimulate lateral root formation and elongation while alleviating ethylene-mediated growth inhibition. The resulting expanded root systems enhance soil water extraction and nutrient uptake, particularly for poorly mobile elements such as phosphorus and iron, thereby improving plant performance under water-limited environments [72]. Collectively, these studies demonstrate that bacterial enhancement of root architecture and nutrient acquisition constitutes a key drought-mitigation mechanism. By promoting deeper, more extensive, and physiologically active root systems, beneficial bacteria improve plant access to water and nutrients, supporting sustained growth and yield under drought stress.

### 3.3. Osmotic Adjustment and Antioxidant Protection

Drought causes cellular dehydration and oxidative stress in plants [81]. Beneficial bacteria help counter these effects via osmotic adjustment accumulating compatible solutes that retain water in tissues and by bolstering the plant’s antioxidant defense system [82,83]. Many drought-responsive PGPB induce higher levels of osmolytes in plants or even contribute their own. Simultaneously, they trigger increased antioxidant enzyme activities and reduce oxidative damage markers. This dual action minimizes drought-induced cellular injury, maintaining membrane integrity and photosynthetic activity [84,85].

Experimental evidence demonstrates that bacterial inoculation consistently promotes osmotic adjustment and antioxidant protection under drought stress. For example, Batool et al. (2020) reported that *Bacillus subtilis* treated potato plants maintained higher soluble sugar and protein contents and showed enhanced CAT, POD, and SOD activities, accompanied by reduced lipid peroxidation and improved growth and tuber yield under water deficit [86].

Similarly, Lee et al. (2024) showed that inoculation of rice with Bacillus megaterium strains increased proline and soluble sugar accumulation and induced the expression of key antioxidant genes (*OsCAT*, *OsAPX*, and *OsSOD*), resulting in higher chlorophyll content and survival rates during drought stress [87].

At the management and signaling level, Zamani et al. (2024) demonstrated that co-application of Bacillus velezensis with salicylic acid synergistically reduced oxidative damage in drought-stressed common bean by lowering H_2_O_2_ and MDA levels while enhancing antioxidant enzyme activities, leading to improved water status and yield [88].

At a broader scale, Shaffique et al. (2022) summarized evidence across multiple crops indicating that PGPR inoculation commonly enhances osmolyte accumulation (e.g., proline and trehalose) and activates antioxidant enzymes, thereby mitigating dehydration-induced oxidative stress [89].

Osmotic adjustment and antioxidant protection represent tightly linked biochemical mechanisms underlying bacteria-mediated drought tolerance. By enhancing the accumulation of compatible solutes and strengthening enzymatic and non-enzymatic antioxidant systems, beneficial bacteria help plants maintain cellular hydration, limit oxidative damage, and preserve photosynthetic function under water deficit. These bacterial-induced adjustments stabilize cellular homeostasis and contribute to sustained growth and productivity during drought stress.

### 3.4. Modification of the Rhizosphere and Soil Structure

Beyond direct effects on plant physiology, drought-adapted bacteria enhance drought tolerance by modifying the rhizosphere environment [90]. Many beneficial bacteria produce exopolysaccharides and form biofilms that stabilize soil aggregates, improve soil structure, and increase water retention around roots [91,92]. These microbial matrices create a hydrated microzone in the rhizosphere, reducing water loss and facilitating root water uptake during drought [92]. In addition, PGPR influence soil porosity and microbial community composition, strengthening root–soil contact and improving soil water-holding capacity under water-limited conditions [93]. Soil organic amendments such as humic substances further modulate microbial community structure and metabolic activity by acting as redox-active compounds and electron shuttles, thereby influencing nutrient cycling and rhizosphere functionality under stress conditions [94].

Experimental and field evidence supports the importance of this mechanism. Ilyas et al. (2020) [95] showed that EPS-producing rhizobacteria, including *Variovorax* and *Achromobacter* spp., improved soil moisture retention and aggregation in wheat rhizospheres, resulting in significantly better plant growth under rainfed drought conditions. Similarly, Khan and Bano (2019) demonstrated that biofilm- and EPS-forming bacteria, particularly when combined with salicylic acid, enhanced leaf water status and grain yield of wheat in drought-prone fields, highlighting synergistic interactions between microbial soil modification and plant stress signaling [96].

At a broader level, reviews have emphasized that EPS-mediated changes in soil structure represent a conserved bacterial strategy for drought mitigation. Bhagat et al. (2021) [97] summarized evidence showing that bacterial EPS improves soil aggregation, water-holding capacity, and nutrient diffusion, thereby sustaining plant growth under water deficit. In a comprehensive review, Tsotetsi et al. (2022) summarized that EPS-producing *Bacillus* spp. contribute to drought tolerance by forming protective biofilms that enhance soil structure, reduce surface crusting, and support root growth and water acquisition under water-limited conditions [98].

Genetic evidence further confirms the central role of exopolysaccharides (EPS) in bacteria-mediated drought tolerance. Lu et al. (2018) demonstrated that an EPS-producing *Bacillus amyloliquefaciens* FZB42 strain significantly enhanced drought tolerance in *Arabidopsis thaliana*, whereas an EPS-deficient epsC mutant failed to confer protection and showed impaired root colonization. These results highlight the importance of bacterial EPS for rhizosphere hydration, biofilm formation, and effective plant–microbe interactions under drought stress [99].

These findings indicate that rhizosphere modification through EPS production and biofilm formation constitutes a critical indirect mechanism of bacteria-induced drought tolerance. By improving soil structure and moisture retention at the root interface, beneficial bacteria create favorable microenvironments that support plant water acquisition and resilience under drought conditions. While EPS-mediated rhizosphere modification plays a particularly important role under severe drought by stabilizing soil structure and maintaining root–soil hydraulic continuity [100,101], phytohormone-mediated effects such as IAA production are often more influential under moderate drought through stimulation of root architectural changes [102,103]. ACC deaminase activity becomes increasingly important under high stress levels by limiting ethylene-induced root growth inhibition [104,105], whereas antioxidant induction mainly mitigates downstream oxidative damage [106,107].

## 4. Types of Beneficial Bacteria Involved in Drought Tolerance

### 4.1. Plant Growth-Promoting Rhizobacteria (PGPR)

Plant growth-promoting rhizobacteria are root-colonizing bacteria that enhance plant growth and stress tolerance through diverse biochemical mechanisms. Under drought conditions, PGPR help plants maintain growth by improving root architecture, modulating phytohormone levels, and inducing osmotic adjustment and antioxidant defenses [108,109]. Many PGPR produce ACC deaminase, which lowers stress-induced ethylene in plants, and synthesize EPS that bind soil moisture, thereby protecting plants from dehydration [110]. As a result, inoculating crops with drought-tolerant PGPR has emerged as a promising strategy to enhance drought resilience [108].

Recent studies demonstrate the considerable benefits of PGPR for drought-stressed plants. A comprehensive meta-analysis reported significant improvements in biomass accumulation, photosynthetic performance, and water-use efficiency under drought conditions, with C4 species exhibiting particularly strong recovery responses [111]. In maize (*Z. mays*), endophytic and rhizobacterial strains have also been reported to enhance stress resilience [112]. Individual case studies corroborate these broad trends. Buqori et al. (2023) reported that inoculation with two *Bacillus* strains producing IAA and ACC deaminase greatly enhanced root length, root branching, and biomass in droughted sugarcane, accompanied by elevated antioxidant enzyme activities (catalase, peroxidase) and lower leaf malondialdehyde levels [109]. Likewise, Sati et al. (2023) reviewed molecular mechanisms of PGPR-mediated drought tolerance and noted that many strains solubilize phosphorus and fix nitrogen to improve plant nutrition, while also triggering stress-responsive genes via phytohormone signaling pathways [113]. Additionally, community-based and multifunctional PGPR approaches have highlighted the importance of complementary traits in enhancing plant performance under stress. Wang et al. (2023) characterized *Acinetobacter oleivorans* S4 as a hydrocarbon-degrading plant growth-promoting rhizobacterium and demonstrated that its inoculation significantly enhanced plant growth while improving contaminant removal efficiency in petroleum-impacted soils [114]. These findings suggest that multifunctional PGPR strains possessing diverse metabolic capabilities can confer additive or synergistic benefits to plants. Collectively, such advances underscore that PGPR—whether applied as single multifunctional strains or strategically assembled consortia—can substantially enhance plant resilience by improving root function, nutrient acquisition, and stress mitigation mechanisms.

### 4.2. Endophytic Bacteria

Endophytic bacteria reside within plant tissues without causing disease and often establish mutualistic associations that can enhance host performance under stress conditions [115]. In the context of drought, certain endophytes have been reported to modulate plant physiology from within, for example by influencing phytohormone balance or enhancing antioxidant capacity. Some endophytes may also be vertically transmitted through seeds, contributing to transgenerational stress adaptation [116]. Owing to their internal localization, endophytes can interact closely with plant tissues and potentially influence processes such as stomatal regulation, nutrient allocation, and stress signaling pathways, thereby contributing to improved tolerance under water deficit.

Multiple recent studies have documented the drought-alleviating impacts of endophytic bacteria across various crops. Jeong et al. (2021) isolated 42 bacterial endophytes from seeds of invasive wild lettuce (*Lactuca serriola*), finding that all exhibited at least one plant growth-promoting trait. Notably, one seed-borne strain (*Kosakonia cowanii*) produced abundant EPS under low water potential and significantly improved *Arabidopsis* growth and soil moisture retention under drought [117]. This suggests endophytes can directly augment the plant’s water-holding microenvironment via polysaccharide production. In crop species, Abideen et al. (2022) showed that endophytic *Pseudomonas* and *Pantoea* strains enhanced drought tolerance in barley (*Hordeum vulgare*) by boosting the plants’ photosynthetic rates and antioxidant defenses. In their experiments, inoculated barley maintained higher chlorophyll content and lower oxidative membrane damage than non-inoculated controls during water stress [118]. Similarly, Juby et al. (2023) reported that two endophytic isolates, *Bacillus safensis* Ni7 and *Stenotrophomonas* C3, significantly promoted growth of pepper (*Capsicum annuum*) seedlings under drought. Treated pepper seedlings showed ~2-fold greater fresh weight and higher relative water content than uninoculated seedlings, along with improved stomatal behavior and chlorophyll levels [119]. These improvements were statistically significant and indicate robust drought mitigation by the endophytes. On a metabolic level, endophytes appear to help plants maintain osmotic balance and scavenge reactive oxygen species: for example, inoculation often elevates proline and sugar levels in host tissues while inducing plant antioxidant enzymes like superoxide dismutase and catalase [115,119]. A recent integrative study on the extremophile succulent *Portulaca oleracea* found that drought caused minimal disruption to its endophytic community, suggesting these drought-hardened endophytes persist and function during stress, and rapid shifts occurred upon rewatering to help recovery [116]. Taken together, such findings illustrate that endophytic bacteria can act as an “internal drought shield,” actively reinforcing the plant’s physiological resilience to water scarcity. By colonizing seeds or vascular tissues, these bacteria impart benefits ranging from improved root growth and nutrient uptake to stress-responsive gene induction and antioxidative protection, ultimately translating into higher drought survival and yield in inoculated plants relative to controls [118,119].

### 4.3. Rhizosphere-Associated Bacteria

Beyond specific inoculant strains (including many PGPR), the broader rhizosphere microbial community—the ensemble of microorganisms inhabiting the soil immediately around roots—plays a pivotal role in plant drought tolerance. Drought stress can alter the composition and function of rhizosphere bacteria, generally selecting for hardy, drought-adapted taxa that can assist the plant in stress adaptation [120,121]. For example, studies have observed that drought tends to reduce overall bacterial diversity in the rhizosphere while enriching certain actinobacteria (such as *Streptomyces* and *Leifsonia* spp.) known for their desiccation tolerance and ability to produce protective metabolites [120,121]. The host plant can actively influence these shifts; under water deficit, plants exude different patterns of root exudates that recruit beneficial microbes capable of enhancing drought resilience [116]. This dynamic feedback leads to a drought-responsive rhizobiome that, in turn, helps the plant by improving soil structure, nutrient availability, and stress signaling. Indeed, Liu et al. (2022) found that drought-sensitive and drought-tolerant cultivars of sugarcane fostered distinct rhizosphere bacterial communities under drought, and the drought-tolerant cultivar’s microbiome showed greater abundance of beneficial genera like *Sphingomonas* and *Bacillus* [122]. Such findings indicate that plant genotype and drought stress together shape a supportive microbial consortium in the root zone.

Harnessing entire rhizosphere communities (rather than single isolates) has become a frontier for improving drought tolerance. Recent advances in microbiome engineering suggest that tailored consortia of rhizosphere bacteria can provide synergistic benefits to plants. In a striking example, Arnault et al. (2024) [123] independently showed that multi-strain bacterial mixtures outperform any single strain in promoting plant growth under drought. These consortia produce a broader spectrum of metabolites (including antibiotics, enzymes, and phytohormones) and more effectively suppress soil-borne pathogens, thereby enhancing stress resilience on multiple fronts [124]. Silverstein et al. (2023) further demonstrated the power of microbiome augmentation: by inoculating “keystone” drought-tolerant species into the rhizosphere, they were able to shift the community structure in favor of beneficial organisms and significantly improve soil fertility and plant water status during drought [125]. These strategic manipulations of the root microbiome mirror natural plant–microbe cooperation. Under field drought, many plants have been observed to accumulate higher populations of symbiotic rhizobacteria (e.g., *Azospirillum*, *Pseudomonas*, *Rhizobium*, *Paenibacillus* spp.) which can help maintain growth. In pearl millet, for instance, Zhao et al. (2023) noted that inoculation with a mix of such PGPR restored drought-stressed plants to near-normal physiological performance, corroborating that a holistic microbial support system can effectively buffer crops against drought [111]. Overall, rhizosphere-associated bacteria function as a critical first line of defense for roots under drought by improving soil structure (via biofilm and EPS production) [110], facilitating deeper root growth and water uptake, and producing drought-alleviating compounds, a healthy rhizosphere microbiota greatly amplifies a plant’s ability to withstand prolonged water shortage. Emerging research on microbiome transplantation and soil inoculants targeting the rhizosphere holds promise for sustainable drought management in agriculture [124].

### 4.4. Cyanobacteria, Mycorrhizal Fungi, and Other Beneficial Microbes

**Figure 3:**
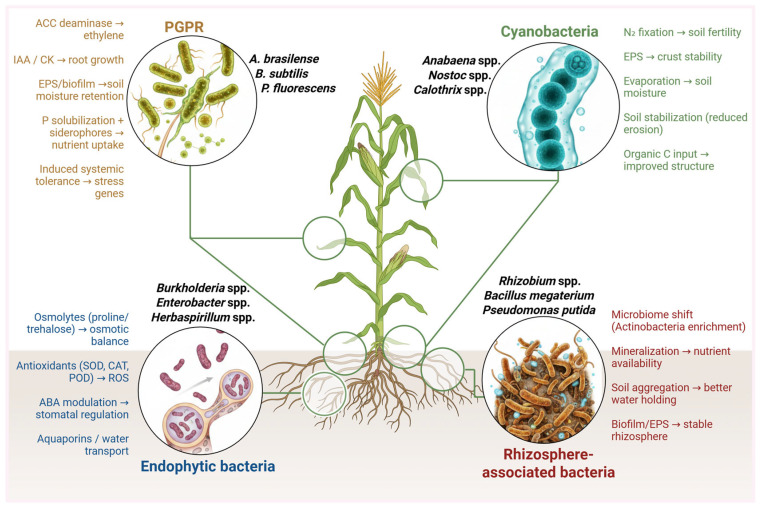
Schematic representation of major groups of beneficial bacteria involved in plant drought tolerance and their primary mechanisms. PGPR colonize the root surface and enhance drought tolerance via ACC deaminase activity, phytohormone production, exopolysaccharide/biofilm formation, and improved nutrient acquisition. Endophytic bacteria reside inside root tissues and support osmotic adjustment, antioxidant defense, and hormonal regulation. Rhizosphere-associated bacteria shape the near-root microbiome, improving soil aggregation, nutrient cycling, and stress resilience. Cyanobacteria contribute mainly at the soil surface by forming biocrusts, fixing nitrogen, producing EPS, and reducing evaporation, collectively improving plant water status and maintaining growth under drought. Created with BioRender.com (2026) by B.D. Kossalbayev (License No. AK29BLYGZ1).

Cyanobacteria photosynthetic bacteria often found in soil and aquatic environments have gained attention as beneficial microbes that can improve plant performance in arid conditions. Together with other beneficial microorganisms, including PGPR, endophytic, and rhizosphere-associated bacteria, cyanobacteria contribute to plant drought tolerance through complementary mechanisms (Figure 3). Recent research underscores the value of harnessing cyanobacterial inoculants for crop drought tolerance. Yadav et al. (2023) [126] showed that inoculating arid soils with a desiccation-tolerant cyanobacterial consortium (*Nostoc punctiforme*, *Scytonema*, and others) accelerated biocrust formation and significantly increased the growth of rice seedlings in dry, nutrient-poor soil. Notably, a mixed cyanobacterial culture provided greater benefits than single-strain treatments, echoing the importance of microbial synergy also seen with PGPR consortia. The cyanobacteria-enriched soil had improved aggregate stability and higher organic carbon, leading to better root development and shoot biomass in rice despite water scarcity [8]. In a similar vein, earlier studies reported that soil inoculation with *Spirulina* (a cyanobacterium) and *Anabaena* enhanced lettuce growth under drought, by maintaining higher leaf relative water content and photosynthetic pigment levels than uninoculated controls. Seed priming with cyanobacteria has also proven effective: Muñoz-Rojas et al. (2018) [127] found that coating seeds of Australian desert plants with *Microcoleus* and *Nostoc* strains greatly improved germination and seedling establishment in dry soils. These outcomes are attributed to the cyanobacteria’s ability to retain moisture and provide nutrients during early plant growth.

Finally, other beneficial microbes such as symbiotic fungi also play a crucial role in drought tolerance. Arbuscular mycorrhizal fungi (AMF), for instance, form mutualistic associations with plant roots and extend the effective root system via fungal hyphae. This not only improves water and mineral uptake but also modulates plant hormonal and antioxidant responses under drought stress. Nader et al. (2024) [128] demonstrated that co-inoculating soybean with AMF and drought-tolerant PGPR bacteria had a synergistic effect, leading to higher shoot and root biomass under drought than either inoculant alone. The AMF enhanced soil water exploitation and delivered nutrients (especially phosphate) to the plant, while the bacteria provided growth hormones and stress-relief metabolites, together ensuring better turgor maintenance and yield protection in water-limited conditions [108]. Similarly, endophytic fungi like *Epichloë* in grasses are known to improve host dehydration tolerance by inducing accumulation of osmolytes and anti-stress alkaloids. Biocontrol fungi such as *Trichoderma* also can augment drought tolerance by stimulating root growth and the plant immune system. In summary, the panorama of beneficial microbes in drought contexts spans multiple domains of life from bacteria (rhizobacteria, endophytes, actinobacteria, cyanobacteria) to fungi (mycorrhizae, endophytes) all contributing in complementary ways. Harnessing these organisms, individually and in combination, is a powerful strategy to fortify plants against drought. By integrating PGPR inoculants, mycorrhizal symbiosis, and even cyanobacterial biofertilizers into crop management, researchers and farmers are developing bio-based solutions to maintain productivity under increasingly erratic rainfall patterns [129].

## 5. Application Strategies of Beneficial Bacteria

### 5.1. Seed Inoculation

**Figure 4:**
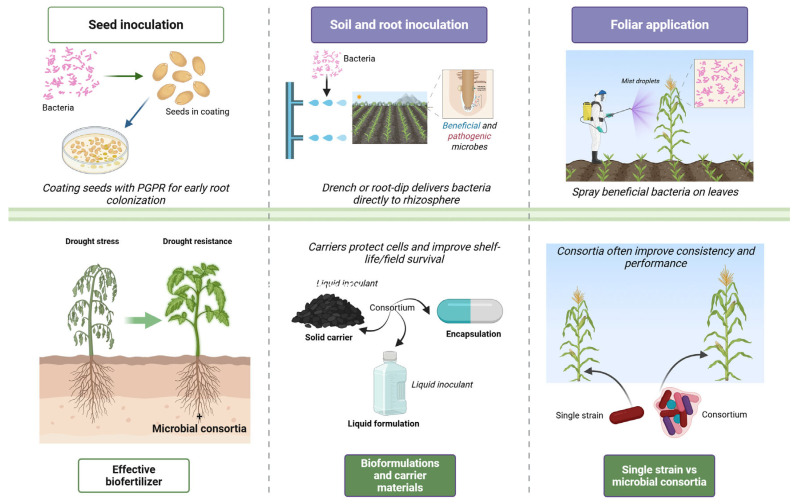
Application strategies and formulation approaches for beneficial bacteria in drought mitigation. Seed inoculation through coating promotes early root colonization; soil and root inoculation via drenching or root-dip methods deliver beneficial bacteria directly to the rhizosphere; foliar application involves spraying bacterial suspensions onto leaves; comparison of single-strain inoculation and microbial consortia highlights enhanced functional stability and performance under drought conditions; bioformulation strategies and carrier materials, including solid carriers, liquid formulations, and encapsulation systems, are designed to improve bacterial survival and field efficacy. Created with BioRender.com (2026) by B.D. Kossalbayev (License No. NW29BLYA3Q).

Seed inoculation involves coating or treating seeds with beneficial microbes prior to sowing (Figure 4), (Table 3). This method ensures that seedlings germinate in the immediate presence of helpful bacteria, promoting early root colonization and vigorous growth. In practice, seeds can be coated with liquid or powdered inoculants using adhesives to help bacteria stick to the seed surface [130]. Benefits of seed inoculation include improved germination rates, seedling vigor, and initial nutrient uptake. For example, a recent review noted that microbial seed coatings with rhizobia, *Azospirillum*, *Bacillus* spp., etc., can enhance seed germination, plant performance, and stress tolerance while reducing the need for agrochemicals [131,132]. In a 2024 study, coating potato seeds with a *Pseudomonas* strain (along with protective polymers) raised germination to 93.5% and produced significantly longer roots and shoots than uninoculated controls, while also suppressing a wilt pathogen [133]. Co-inoculating seeds with multiple microbes and nutrients can yield synergistic effects. For instance, combining rhizobia and mycorrhizal fungi on bean seeds, together with micronutrient foliar feeds, led to greater increases in bean yield and protein content than any single input alone [134]. Seed inoculation is especially established in legumes *Bradyrhizobium* on soybean to ensure nitrogen-fixing nodulation, but it is increasingly applied to cereals and vegetables. A field trial on soybean found that inoculating seeds produced higher nodule numbers, plant dry mass, and yield, highlighting the efficiency of seed delivery [135]. Similarly, in tomato, seed/seedling inoculation with *Bacillus subtilis* and *B. amyloliquefaciens* increased productivity, treated plants bore ~76% more fruits per plant and ~33–50% larger fruits than controls [136]. These examples demonstrate that seed inoculation is a practical, cost-effective strategy to introduce beneficial bacteria at the very start of the plant’s life cycle, often leading to improved crop growth and yield outcomes.

### 5.2. Soil and Root Inoculation

Soil inoculation refers to applying beneficial bacteria directly into the soil so that they colonize the rhizosphere, whereas root inoculation often means treating young plant roots. These methods aim to establish a high population of PGPR around or on roots in situ. Soil inoculants can be delivered as powder, granules, or water-based drench. For instance, farmers may mix dry bacterial formulations into the soil at planting or use liquid soil drenching to water in the bacteria around seeds or root zones. An advantage of soil inoculation is that it can be done for existing plants and can cover a broad root area. A 2024 study on blueberry used a root zone irrigation with a consortium of PGPR; treated plants showed significantly greater branch and leaf growth, and the surrounding soil had higher nutrient availability and a more diverse microbial community than untreated soil [137]. Soil inoculation is also used to combat soil-borne diseases by introducing biocontrol bacteria into the soil. Abdel-Kader et al. (2023) tested soil-drenching wheat with *Bacillus* and *Pseudomonas*, followed by foliar spraying, to suppress foliar diseases. They observed that plots receiving beneficial microbes had lower disease severity and correspondingly higher grain yields than untreated plots [138]. This illustrates how soil application can improve plant health via both nutritional and protective effects. However, soil inoculation may require larger inoculum quantities than seed coating, and environmental factors can influence efficacy [139]. Interestingly, the method of inoculation can make a difference: in a soybean trial, seed inoculation outperformed soil application in boosting nodulation and plant biomass [135]. Soil and root inoculation strategies are versatile and can be timed at planting or during crop growth, often used in tandem with organic amendments or as a part of integrated nutrient management to enhance soil fertility and crop performance.

### 5.3. Foliar Application

Foliar application involves spraying beneficial microbes onto the leaves and above-ground parts of plants. While most PGPR colonize roots, certain phyllosphere-competent bacteria can survive on leaf surfaces and confer benefits such as induced disease resistance or growth promotion. Foliar sprays deliver microbes directly to sites of pathogen entry on leaves and can trigger systemic resistance in the plant. This method is often used where soil application is impractical or as a complementary treatment. For example, farmers growing leafy greens have applied PGPR via foliar feeding to improve growth: one study reported that a *Pseudomonas* bioformula increased lettuce yields by 10–20% when applied as foliar spray, even under reduced fertilizer regimes [140]. Foliar-applied PGPR can also help plants tolerate stress. Certain strains produce osmoprotectants and antioxidants on leaf surfaces that enhance drought or heat tolerance when absorbed through stomata [141]. The most documented use of foliar microbial application is in biocontrol of foliar diseases. For instance, spraying wheat leaves with *Trichoderma* or *Pseudomonas fluorescens* significantly reduced powdery mildew and rust infection, which corresponded with higher grain yields compared to unsprayed controls [138]. Likewise, foliar spray of a mix of *Bacillus* spp. on tomato was found to increase leaf thickness and chlorophyll while suppressing leaf spot disease, ultimately improving tomato fruit yield and quality [136]. Foliar application is generally considered “where applicable,” because not all beneficial microbes can survive the UV exposure and desiccation on leaf surfaces. Formulations for foliar sprays often include protective agents to help microbes adhere to and persist on the foliage [130]. Another consideration is that timing of foliar application is critical it is often done during cooler times of day or in humid conditions to prolong leaf wetness for microbial activity. When used appropriately, foliar inoculation can serve as a rapid intervention to boost plant health and is a useful complement to soil/seed inoculation. In practice, a combined approach is beneficial: in pinto beans, seed inoculation plus strategic foliar feeding produced the best gains in yield and seed quality, demonstrating the value of integrating foliar applications with other methods [134].

### 5.4. Single Strains vs. Microbial Consortia

Beneficial microbes can be applied as single-strain inoculants or as multi-strain consortia. Early biofertilizers often used a single effective strain. However, research in the past decade has shown that combining complementary microbes can broaden the range of benefits and enhance consistency of performance [142]. Mixed consortia may include bacteria with different functional traits that work in synergy to benefit the plant. For example, one field study on teff grain reported that a three-strain PGPR consortium increased grain yield by ~12% compared to uninoculated control, outperforming individual inoculations, and improved nutrient uptake in the [142]. The combined-inoculum plants were taller and higher-yielding than those receiving any single strain. In another case, a consortium of five bacteria as *Azotobacter*, *Bacillus*, *Paenibacillus*, *Pantoea*, *Pseudomonas* were tested in semi-arid maize fields, soil health indicators improved, and the optimal consortium dose raised grain yield significantly more than lower doses or single-strain treatments [13]. Laboratory and greenhouse experiment also confirm the synergistic interactions in consortia. Wang et al. (2025) showed that co-inoculating tomato with two specific strains like *Pantoea* D1-28 + *Bacillus* LAD led to biofilm formation on roots and a dramatic boost in growth, tomato shoot, and root biomass increased two- to five-fold, much higher than with either strain alone [135]. Such synergy is often attributed to one microbe creating a favorable environment that allows the other to thrive and jointly enhance plant growth [143]. That said, not all combinations work; strains must be compatible and not antagonistic to each other. Careful screening is needed to ensure consortium members complement rather than inhibit each other [141,142]. When well-formulated, multi-microbe products have shown superior performance. A recent analysis found that on average, a multi-strain PGPR inoculant improved plant biomass by ~48%, compared to ~29% improvement by the best single-strain inoculant in the same system. Consortia can also provide more consistency across environments because different microbes may perform under different soil conditions, thus buffering the variability often seen with single-strain biofertilizers [144,145]. On the other hand, single strains are easier to quality-control and study. In practice, the choice might depend on the target outcome, a specific pathogen biocontrol might use a single highly antagonistic bacterium, whereas a biofertilizer for yield enhancement might combine a nitrogen fixer, a P-solubilizer, and a phytostimulator. Overall, the trend is toward consortium inoculants as research continues to demonstrate their broad and sometimes synergistic benefits over single strains [142,146].

### 5.5. Bioformulations and Carrier Materials

Developing effective bioformulations is key to delivering beneficial bacteria in practical agricultural settings. These formulations include the living microbial strain plus any carrier or additive that helps maintain viability and facilitate application. A good carrier provides a protective environment for the bacteria during storage and after application [130]. Traditional solid carriers are peat-based, as peat has an excellent moisture-holding capacity and contains organic matter that supports bacterial survival. Peat has long been called the “gold standard” for rhizobial inoculants. Other common carriers include talc powder, vermiculite, charcoal, compost, and soil mixtures [130]. These materials are inexpensive and can be sterilized and amended with nutrients to support the microbes. For instance, sterile cocopeat has emerged as a popular carrier; it is biodegradable, holds water well, and hasbeen shown to support high survival of inoculant bacteria and good root colonization [147]. In one study, a cocopeat-based formulation was used to coat tomato seeds; it significantly improved seedling vigor and root development compared to non-formulated inoculum [147]. Liquid formulations are also widely used here bacteria are delivered in a liquid broth or oil-based suspension. These often include osmoprotectants to prolong shelf-life. Carrier selection greatly affects shelf-life of the biofertilizer. Researchers found that carriers with high water retention and balanced nutrients allowed bacteria to remain viable for longer periods than drier, carbon-rich carriers [130]. Additionally, adding certain polymers as adjuvants can improve formulation quality, alginate, starch, or cellulose derivatives are used to encapsulate cells or as stickers. Encapsulation in alginate beads provides an excellent microenvironment; remarkably, dried alginate bead inoculants have kept some bacteria alive for years in storage [130]. Newer techniques are exploring nanomaterials and biofilm-based formulations. Nano-encapsulation can shield PGPR from heat and UV, thereby increasing stability and efficacy [141]. For example, researchers have developed chitosan-alginate nano-capsules that release bacteria gradually and improve their stress tolerance on crop leaves [148,149]. Another innovation is biofilm inoculants: one 2025 study created a biofilm of cyanobacteria plus two bacterial strains, then applied this as a biofertilizer capsule in rice. The biofilm matrix enhanced microbial survival in the field and, with 25% reduced chemical fertilizer, the biofilm-treated rice matched or exceeded normal yields [150]. Such formulations exemplify next generation bioinoculants that aim to address past consistency issues. Finally, the physical form of a bioformulation is tailored to application method: peat or clay powders for seed coating, granules for soil application, and liquids for drip irrigation or foliar spray. To ensure quality, many countries have set standards for carrier-based products. In summary, advances in formulation science from better carrier materials to protective encapsulation and biofilm techniques are making beneficial bacteria products more robust and farmer friendly [150]. These bioformulations and carriers are crucial for delivering the promise of PGPR from the lab to the land in a reliable way.

**Table 3:** Overview of application strategies for beneficial microorganisms in agriculture.

Strategy	Description and Method	Key Advantages	Challenges/Considerations	Ref.
Seed inoculation	Coating seeds with beneficial bacteria. Typically done just before sowing; bacteria adhere to seed surface and colonize the emerging root.	Early root colonization right at germination; improved seedling vigor, germination rate, and nutrient uptake from emergence; low cost and easy to implement.	Bacteria must survive on seed until planting; high seed rates or seed storage in harsh conditions can reduce viability of inoculant; adhesion to seed sometimes requires stickers and uniform coating for best results.	[130,132,133]
Soil and root inoculation	Applying inoculant directly into soil or onto roots. Methods include soil drenching with liquid biofertilizer, mixing dry granules or powder into soil, or dipping transplant roots in bacterial suspension.	Treats the rhizosphere soil volume, potentially reaching more root area than seed coat; can be done after plant establishment for supplementary inoculation; useful for delivering microbes that protect roots from soil-borne pests/pathogens in situ.	Soil environment can be harsh; often requires larger quantities of inoculum per hectare than seed treatment; application must ensure good contact with root zone; effectiveness can vary with soil type, moisture.	[139]
Foliar application	Spraying a suspension of beneficial bacteria on crop foliage. Often done using a sprayer at a certain growth stage. Microbes land on leaf surfaces, and some may colonize the phyllosphere or enter via stomata. Sometimes combined with nutrient sprays.	Can be applied when needed for more immediate effect; some strains induce systemic resistance, helping the plant fight foliar pathogens; can be integrated into regular foliar feeding or crop protection schedules.	Not all beneficials survive UV light, drying, and other surface conditions on leaves; requires good coverage on foliage for efficacy; rain or irrigation can wash off the inoculant from leaves; leaf surface wettability and cuticle thickness may limit bacterial adhesion and penetration.	[138]
Single-strain vs. consortium	Choice of using one microbial species/strain versus a mix of multiple strains. *Single-strain* inoculant: pure culture of *Azospirillum brasilense*. *Consortium*: formulated blend of 2–5 PGPR species.	Consortia can offer synergistic effects: multiple mechanisms; if one strain fails in certain soil, another may still thrive, giving more consistent benefits; can target diverse issues simultaneously.	Multi-strain mixes need compatibility: strains may inhibit each other or compete if not well-matched; regulatory approval can be more complex; single strains are simpler to mass-produce and quality-control.	[142]
Bioformulation and carriers	The format in which the microbial product is delivered. Includes solid carriers, liquid formulations, and novel encapsulations. Often contain additives: protectants, nutrients, or adhesives.	Proper formulation greatly extends shelf-life and field viability of the microbes; carriers like peat, cocopeat, or biochar provide moisture and nutrients to inoculant, boosting survival after application.	Formulation adds cost–requires specialized production; storage conditions still important: high temperature or direct sun can kill inoculants even in good formulations.	[130]

## 6. Challenges and Limitations

*Inconsistent field performance*. Despite strong greenhouse and growth-chamber results, bacterial inoculants often show variable or even negligible benefits under real field conditions [151]. This lab-to-field gap is driven by strong spatial/temporal heterogeneity in soil properties (pH, texture, organic matter, salinity), climate variability [152], and management practices (fertilizer regime, pesticides, irrigation, tillage) that can either suppress the inoculant or mask its effect. In addition, introduced strains must establish and function within complex resident microbiomes; antagonism, predation, nutrient competition, and priority effects can prevent the inoculant from reaching an effective population size [153]. Consequently, the same strain may work well in one location/season but fail in another, reducing farmer confidence and slowing adoption [154].

Concrete case studies further illustrate this lab-to-field discrepancy. Several reviews and field evaluations report that strains performing consistently under greenhouse conditions may show diminished or unstable responses across field sites due to soil heterogeneity and microbial competition [13,155]. In addition, successful colonization observed in controlled substrates is not always maintained under open-field drought conditions, where environmental filtering and native microbiome interactions limit population stability [156]. These findings highlight that ecological fitness and multi-location validation are critical determinants of field efficacy.

*Survival and colonization under stress.* A core limitation is that many beneficial bacteria do not survive storage-to-field transitions or fail to colonize roots consistently under drought and related stresses. During storage and handling, cells may lose viability; after application, they experience rapid desiccation, osmotic shock, temperature extremes, nutrient limitation, UV exposure, and biotic pressures from native microbes [157]. Even when cells survive, effective colonization is not guaranteed: root attachment, biofilm formation, chemotaxis toward exudates, and persistence over time can be disrupted by low soil moisture and by competitive exclusion from established communities. In symbiotic systems, weak competitiveness for infection/nodule occupancy and poor persistence across seasons are well-known bottlenecks, illustrating why excellent strains may still underperform in farmers’ fields [151,157,158].

*Host specificity and environmental factors.* Beneficial effects are frequently host- and context-dependent. Plant genotype influences root exudate chemistry, immune signaling, and niche availability, shaping which microbes can colonize and deliver benefits. This means a strain optimized on one cultivar or in one soil may not translate to another cultivar or agroecological zone [154]. Environmental filters, soil pH, nutrient status, moisture history, temperature, and background microbiome composition also interact with the host to determine establishment and function [157]. Evidence from controlled and semi-controlled studies shows that genotype and inoculation regime can significantly alter outcomes, reinforcing the need for crop-strain matching and multi-site validation rather than one-size-fits-all products [158].

*Regulatory and commercialization issues.* Commercial deployment faces additional constraints beyond biology. Regulatory definitions and requirements vary across regions, and there can be mismatches between legal lists of permitted microorganisms and updated phylogenetic/systematic classifications. Standardization challenges include strain identity verification, viable counts at shelf-life, contamination control, and consistency across batches, issues that directly affect efficacy and safety [159]. From a market perspective, developers must also navigate intellectual property, scale-up/fermentation costs, formulation stability, distribution logistics, and performance claims that regulators may restrict unless supported by robust field datasets [160]. Recent reviews emphasize that clearer regulatory alignment, harmonized quality standards, and realistic claims linked to defined use-conditions are essential to accelerate responsible commercialization [161,162]. It is important to acknowledge potential publication bias in the current literature, as most published studies report positive effects of PGPR on drought tolerance, whereas non-significant or inconsistent results are less frequently documented. Field evaluations and commercialization analyses have highlighted variability and context-dependent efficacy that is not always reflected in controlled studies [13,155]. This imbalance may lead to an overestimation of efficacy and underscores the need for transparent reporting and multi-site validation trials.

## 7. Conclusions

Beneficial bacteria represent a powerful and versatile resource for enhancing plant drought tolerance in a sustainable and environmentally friendly manner. Accumulating evidence demonstrates that diverse microbial groups including PGPR, endophytes, rhizosphere-associated bacteria, and cyanobacteria contribute to drought resilience through interconnected mechanisms operating at the plant, root, and soil levels. These microorganisms regulate plant hormonal balance, reduce stress-induced ethylene accumulation, improve osmotic adjustment, enhance antioxidant capacity, and facilitate nutrient acquisition, while simultaneously improving soil aggregation, moisture retention, and microbial community stability. Importantly, these effects are not isolated but act synergistically to maintain plant growth and productivity under water-limited conditions. Beyond microbial functionality, application strategy plays a decisive role in determining the success of bacterial inoculants in agricultural systems. Seed, soil, root, and foliar inoculation methods each offer distinct advantages depending on crop type, growth stage, and environmental context. Increasingly, multi-strain microbial consortia are demonstrating superior and more consistent performance than single-strain inoculants, reflecting functional complementarity and ecological robustness. Parallel advances in bioformulation science particularly the development of optimized carrier materials, encapsulation technologies, and biofilm-based inoculants are addressing long-standing challenges related to microbial survival, shelf-life, and field reliability. Despite substantial progress, several challenges remain. Field performance of microbial products can be influenced by soil type, climate variability, crop genotype, and interactions with native microbiota. Future research should prioritize multi-location field trials, systems-level analyses of plant–microbe soil interactions, and the rational design of tailored microbial consortia. Integrating beneficial bacteria with conventional agronomic practices, precision agriculture, and climate-smart management strategies will be essential to fully realize their potential. Overall, harnessing beneficial bacteria offers a promising pathway toward resilient, low-input agricultural systems capable of sustaining productivity under increasingly frequent and severe drought conditions.

## Figures and Tables

**Table 1:** Major transcription factor families involved in plant drought stress responses and their regulatory roles.

Transcription Factor Family	Representative Transcription Factors	Major Regulated Processes/Target Genes	Ref.
AP2/ERF (DREB subfamily)	*DREB1A*, *DREB2A*	Dehydration-responsive genes; LEA proteins, dehydrins, osmolyte biosynthesis	[41,42]
bZIP	*ABF1*, *ABF2*, *AREB1*	ABA-dependent signaling; antioxidant enzymes; stress gene expression	[43,44]
NAC	*SNAC1*, *NAC6*	Stress-induced growth regulation; survival and senescence pathways	[45]
WRKY	*WRKY33*, *WRKY46*	Redox balance; antioxidant and stress signaling	[46,47]
bHLH	*bHLH122*	ABA-responsive gene regulation; drought tolerance modulation	[48,49]

**Table 2:** Beneficial bacteria influencing root architecture and hormone-mediated root responses under drought.

Beneficial Bacterium	Host Plant	Root Architectural Effects Under Drought	Effect Magnitude (%)	Nutrient and Water-Related Outcomes	Key Mechanism for Root Stimulation	Ref.
*Bacillus subtilis*	Sugarcane (*Saccharum officinarum*)	Increased root length, root biomass, and tiller number	Root length ↑ ~20%; Root DW ↑ ~22%; Photosynthesis ↑ 40–67%; WUE ↑ ~40%; Stalk DW ↑ 18–26%; Leaf N ↑ 20–33%	Enhanced N and P uptake, improved WUE, higher sucrose yield	Auxin-mediated root elongation	[69]
Native *Bacillus* spp. (co-inoculation)	Chili pepper (*Capsicum annuum*)	12–16% increase in root surface area and volume	Survival ↑ 4.17%; Water-use efficiency ↑ 185.10% under 0% irrigation	Increased uptake of N, P, Zn, Mn, Cu; improved survival	Root branching stimulation	[70]
Drought-tolerant PGPR (multiple strains) + biochar (BC)	Wheat	Root length/architecture improved under drought (reported as key response)	Root length ↑ 26–34%; Root FW ↑ ~29%; Shoot DW ↑ ~22%; RWC ↑ ~18%; Grain yield ↑ ~21%; MDA ↓ ~27%	Higher RWC and growth-related traits under drought	PGPR-driven root remodeling + BC synergy	[71]
PGPR (*Bacillus*, *Pseudomonas*, *Azospirillum*)	Wheat, maize	Enhanced lateral root formation and elongation	Biomass ↑ 44–50%; RWC ↑ 24%; Proline ↑ 57%; MDA ↓ 28%	Improved soil water extraction and P/Fe uptake	Auxin production and ethylene modulation	[72]
*Pseudomonas fluorescens* YX2	Apple (*Malus domestica*)	Increased root length and root activity	Root activity ↑ 13% (LD), ↑ 26% (MD); soil available P ↑ 16% (LD), ↑ 39% (MD); soil alkaline phosphatase ↑ 21% (MD); PGPR P contribution rate reached 26.25% (MD); total root length increased to 503 cm under LD	Improved phosphorus acquisition	Root growth promotion and P solubilization	[73]
*Bacillus megaterium* (drought-tolerant isolate)	Wheat	Root colonization + improved seedling vigor traits (incl. root-related growth) under drought	↑ RWC (~12–18%); ↑ proline (~20–35%); ↑ chlorophyll (~10–15%); ↓ MDA (~25–30%) under drought vs. non-inoculated	Higher RWC, pigments, osmolytes; reduced MDA	IAA + ACC deaminase + stress-protective responses	[74]
ACC deaminase *Pseudomonas fluorescens* strains	Sweet corn	Ethylene reduction supports root growth potential (ethylene inhibition lowered)	Increased nodule number and plant biomass (exact % not specified)	Better chlorophyll and yield under limited irrigation	ACC deaminase → ↓stress ethylene	[75]
Endophytic bacteria (single/co-inoculum, e.g., *Bacillus* spp.)	Jerusalem artichoke	Root traits (length/surface/volume) improved under drought	Yield ↑ 52–100% (drought) Inulin ↑ 16–24%	Better growth/yield traits under drought	Endophyte-driven growth regulation	[76]
*Pseudomonas fluorescens* G20-18 (cytokinin-producing)	Tomato	Root-related priming + drought preparedness (study focuses on hormone priming; can be used in Section 3.1)	~7-fold increase in drought-responsive DEGs; 85% reduction in DEGs (CNT1 mutant)	Improved drought responses (ABA/stomata/chlorophyll)	Bacterial cytokinins → hormone signaling	[77]
Drought-tolerant *Enterobacter*/*Leclercia* (example strain PAB19)	Mung bean	Improved growth under PEG-drought (can be linked to root vigor/biomass in paper)	Biomass ↑ ~30%; Proline ↑ 40–60%; SOD ↑ 32–45%; MDA ↓ 20–35%	Better plant performance under water deficit	IAA/PGP traits (strain-based)	[78]
*Pseudomonas* spp. + Bacillus sp. consortium (seed bioinoculation)	Maize	Increased root length under drought (explicitly reported)	Chlorophyll ↑ 14.9–15.9%; Soil moisture ↑ 28.7–30.7%; Root length ↑ (10.3 cm vs. control)	Better survival/biomass traits	Consortium effect (PGPR synergy)	[79]
*Azospirillum brasilense* + agronomic practice	Wheat	Improved growth physiology under drought (root-related interpretation possible, paper is drought-focused)	Biomass ↑ 24–32%; RWC ↑ 19%; Proline ↑ 50%; MDA ↓ 28%	Better growth/soil health under drought	PGPR + management synergy	[80]

Note: Arrows indicate the direction of change relative to drought-stressed control plants (↑ increase; ↓ decrease; → no significant change).

## Data Availability

No new data were created or analyzed in this study. Data sharing is not applicable to this article.

## Abbreviations

ABA	Abscisic acid
ACC	1-aminocyclopropane-1-carboxylate
AMF	Arbuscular mycorrhizal fungi
APX	Ascorbate peroxidase
IAA	Indole-3-acetic acid
MDA	Malondialdehyde
NCED	9-cis-epoxycarotenoid dioxygenase
PGPB	Plant growth-promoting bacteria
POD	Peroxidase
ROS	Reactive oxygen species
RWC	Relative water content
SOD	Superoxide dismutase
